# Fused Regression for Multi-source Gene Regulatory Network Inference

**DOI:** 10.1371/journal.pcbi.1005157

**Published:** 2016-12-06

**Authors:** Kari Y. Lam, Zachary M. Westrick, Christian L. Müller, Lionel Christiaen, Richard Bonneau

**Affiliations:** 1 New York University, New York, New York, United States of America; 2 Simons Foundation, New York, New York, United States of America; University of Cambridge, UNITED KINGDOM

## Abstract

Understanding gene regulatory networks is critical to understanding cellular differentiation and response to external stimuli. Methods for global network inference have been developed and applied to a variety of species. Most approaches consider the problem of network inference independently in each species, despite evidence that gene regulation can be conserved even in distantly related species. Further, network inference is often confined to single data-types (single platforms) and single cell types. We introduce a method for multi-source network inference that allows simultaneous estimation of gene regulatory networks in multiple species or biological processes through the introduction of priors based on known gene relationships such as orthology incorporated using fused regression. This approach improves network inference performance even when orthology mapping and conservation are incomplete. We refine this method by presenting an algorithm that extracts the true conserved subnetwork from a larger set of potentially conserved interactions and demonstrate the utility of our method in cross species network inference. Last, we demonstrate our method’s utility in learning from data collected on different experimental platforms.

## Author Summary

Gene regulatory networks describing related biological processes are thought to share conserved interaction structure. This assumption motivates a great deal of work in model systems–where discovery of gene regulation may be more experimentally tractable–but is difficult to directly evaluate using existing methods. The presence of shared structure in a well studied model system or process should make the problem of network inference in a related process easier, but this information is not often applied to the discovery of global gene regulatory networks. Further, to be able to successfully translate findings between different organisms, it is important to be able to identify where regulatory structure is different. We provide a method based on penalized fused regression for inferring gene regulatory networks given prior knowledge about the similarity of interactions in each network. This method is demonstrated on synthetic data, and applied to the problem of inferring networks in distantly related bacterial organisms. We then introduce an extension of the method to deal with the condition of uncertainty over the degree of regulatory conservation by simultaneously inferring gene conservation and interaction weights.

## Introduction

As the volume and variety of genome scale data continues to increase in quantity and quality, the goal of accurately modeling gene regulatory networks has become attainable [1–3]. Large-scale data collection efforts have contributed to the development of high quality networks which accurately represent biological processes, but most processes and organisms remain uncharacterized at the network level. Furthermore, as new technologies are developed and some old ones are replaced, such as RNAseq and microarray, it becomes important to be able to combine data from multiple platforms, lest we lose valuable information from existing studies. The problem of inferring related—but not necessarily identical—structure from related—but not identical—data is ubiquitous in biology. Multi-source network inference has applications for learning multiple networks in related species, for learning networks associated with distinct processes within the same species, and for learning networks based on heterogeneous data sources. Moreover, as it becomes possible to learn genome-wide regulatory networks, we can begin to compare and to test whether there is conservation of networks across species and biological processes. Our use of model organisms to study biological processes and diseases relevant to humans relies on the assumption of conservation; yet this has not been effectively tested at the genome scale.

We present two methods for network inference based on linear estimates of gene expression dynamics, extending existing dynamical-systems methods for network inference [1, 4, 5]. The core of both methods is the observation that biological information about the relatedness of genes can be used to select which network coefficients should be similar to one another in a multi-source network inference problem (ie orthologous TFs should regulate orthologous genes), and that these constraints can be efficiently represented as penalties in a least-squares regression problem.

Numerous studies have shown that functional conservation exists in gene regulatory networks even across large evolutionary distance [6–9]. Our first method—fused L2—takes advantage of this similarity by imposing an L2 penalty on the differences between *a priori* similar interactions (termed fusion penalty). These constraints favor network configurations in which orthologous genes have similar regulators. Because network inference problems are typically under-constrained, these additional constraints allow data in one species to improve network inference performance in another.

Existing multi species approaches often use orthology as a proxy for functional conservation [10–14], or attempt to learn functional similarity via expression data [15]. Orthology can be approximated using readily identifiable sequence similarity, which is often a useful predictor of functional similarity [16, 17]. However, many genes will have evolved different functions and therefore may have new regulatory interactions. For example, gene duplications may lead to neofunctionalization [18] of the duplicated genes. Or, when comparing regulation across cell lines, changes in chromatin configuration may affect our hypotheses about the similarity of interactions between pleiotropic TFs and target genes across cell types (a within-species analog to neo-functionalization) [19].

Identifying interactions that are present in one species but not another is of direct biological interest, but existing approaches to network inference are unable to effectively test the hypothesis of conserved subnetworks. Observing a large difference in the weights of regulatory interactions obtained though independent inference of multiple networks is perhaps the best (least biased) evidence against conservation of orthologous regulatory interactions (cases where target and regulator have orthologs across species). However, this is sometimes weak evidence, as network inference is typically under-constrained [20], meaning there could be a different set of networks for which conservation does hold, and which fit the data almost as well. Solving the networks jointly with fusion addresses this problem, but may be biased when gene function is not conserved.

Our second method—adaptive fusion—attempts to solve the problem of identifying evolutionary divergence using a non-concave saturating fusion penalty to simultaneously infer the constrained networks and to learn which constraints should be relaxed (ie which parts of the network are genuinely different). This penalty is based on statistical techniques intended to provide unbiased regularization penalties for regularized regression [14, 21]. We extend these techniques fused regression, and provide an algorithm that approximates the solution to the resulting non-convex loss function.

We develop two algorithms for solving efficiently multi-output least-squares regression problems with pairwise L2 fusion penalties on entries of the coefficient matrix, and discuss conditions under which each is suitable. We also introduce—in the form of adaptive fusion—the idea of a saturating penalty function on fusion constraints, and estimate the solution to the resulting optimization problem through iterative application of the fused L2 algorithm. We start with a discussion of the fused L2 and adaptive fusion algorithms, and describe their performance on synthetic datasets intended to represent related gene regulatory networks. We then demonstrate the applications of the fused L2 network inference algorithm on biological data, first demonstrating gains in cross-species network inference using the bacteria species *Bacillus subtilis* and the distantly related *Bacillus anthracis*, then moving on to multi-platform network inference using *B. subtilis*, and finally intra-species fused network inference using priors based on operons in *B. subtilis*. We then discuss adaptive fusion and show the technique’s ability to identify incorrect orthology information which has been introduced to a biological dataset, suggesting the technique’s applicability to discovering neo- and sub-functionalizations.

## Results

### Simulations

#### Using fused regression to learn related networks

**Fig 1:**
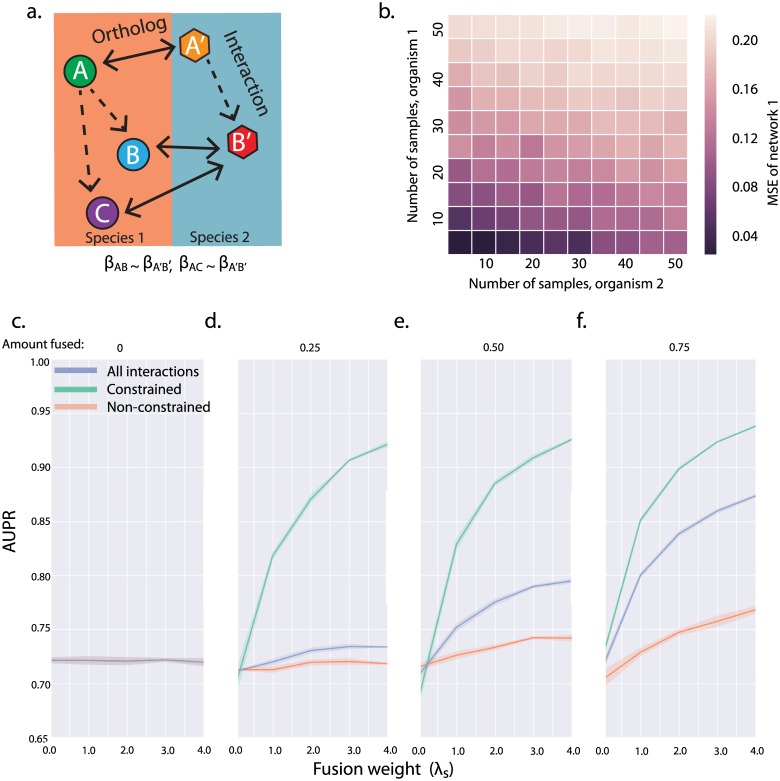
**A.** Schematic representation of the the generation of fusion constraints from orthology mappings. Dashed arrows indicate potential regulatory interactions, while solid arrows denote orthology. We introduce fusion constraints for pairs of interactions for which both the regulator and regulated gene are orthologs of one another. In this example, we would introduce a constraint between the (*A*, *B*) and (*A*′, *B*′) interactions and the (*A*, *C*) and (*A*′, *B*′) interactions. **B.** In order to demonstrate the utility of fused network inference in combining data, we generate two networks with 10 TFs and 200 genes, 75% of which are orthologous (75% sparsity). Mean squared error of the inferred vs. true coefficient matrices for network 1 are plotted as a function of the number of conditions generated for species 1 (x-axis) and the number of conditions generated for species 2 (y-axis). As expected, increasing the number of samples available for the species of interest improves network inference performance. However, because we are fusing to data from a related species, similar gains are observed when increasing the amount of data available in this second species. **C-F** Show the varying effects of fusion on simulated networks with different levels of conservation. We generate a series of networks with 20 TFs by 200 genes in two species, (50% sparsity) while varying the fraction of gene orthologies in the simulated networks (Amount fused, shown above each figure). For each network, we evaluated AUPR on one of the species for: all interactions (blue line), interactions with fusion constraints (green line), and interactions without fusion constraints (orange line). At every level of conservation, constrained interactions show the largest benefits of fusion, with the magnitude of the benefit growing with fraction of orthologous genes. When the networks are highly conserved, however, even interactions that are not directly constrained through fusion are recovered more accurately as λ_*S*_ increases.

We created synthetic networks to approximate two related biological processes, then evaluated performance of our fused L2 regression, which learns the networks simultaneously given a prior on the relatedness of interactions. Simulated networks consisted of pairs of related 10 TF by 200 gene networks, with varying numbers of expression data samples made available to the solver. In order to compare the relative contribution of data in the species of interest (species 1) and in a related organism (species 2) to network recovery, we varied the number of conditions in each species while evaluating the mean-squared-error between the recovered network for species 1 and its known true network. When the amount of data from the related species was held constant, increasing the amount of data available for learning the network for the species of interest resulted in a more accurate network prediction, as expected (Fig 1b). Similarly, when we increased the amount of data from the related species, we obtained performance gains on network one using fused L2 regression, demonstrating our ability to improve network inference on one dataset through incorporation of a related dataset.

#### Fused regression improves performance on both the constrained and non-constrained parts of the network

Our approach is useful for learning networks from similar sources such as related cell types from the same species, where there exists a one-to-one mapping of genes, as well as datasets where the orthology mapping does not span all genes. This latter case is typical not only for cross-species network inference, but can occur when using different technology, eg microarray and RNAseq, where there is incomplete overlap in the genes that each method assays as well as incomplete overlap in the genes expressed in different experimental designs. When orthology is incomplete we are interested in knowing if performance gains from fused regression are limited to those interactions which have fusion constraints, or if they extend to the entire network. To test this we used multiple 20 TF by 200 gene synthetic networks with varying proportions of orthologous TFs and genes. We divided networks into those interactions with fusion constraints (the constrained subnetwork) and interactions without fusion constraints (the non-constrained subnetwork). We varied the weight on the fusion penalty, λ_*S*_, and evaluated performance by computing AUPR on the constrained subnetwork, the non-constrained subnetwork, and the whole network (Fig 1c–1f). Because the conserved subgraphs were known to be similar to each other, we expected performance to improve as the fusion penalty weight increased (Fig 1c–1f, Constrained).

Interestingly, performance gains were seen even in the portion of the network that was unconstrained by fusion (Fig 1c–1f, Non-constrained). This is because a gene may have some interactions that are constrained by fusion—regulation by TFs with orthologs—and some interactions that are unconstrained—regulation by TFs without orthologs. Because both constrained and unconstrained components compete to explain the same pattern of gene expression, improving recovery of the constrained sub-network will tend to improve recovery for the unconstrained sub-network as well.

#### Adaptive fusion with simulated data

**Fig 2:**
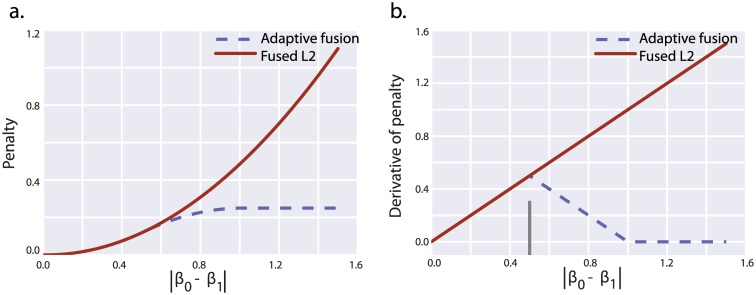
Adaptive fusion loss function (A) and derivative of loss function (B). **A.** Adaptive fusion is a quadratic around the origin, begins to taper at *a*/2, and plateaus at *a*. After the plateau, increasing the difference in interaction weight of fused interactions does not further affect the penalty incurred through fusion. As a result, interaction weights in this zone are effectively unfused from one another (the fusion penalty behaves like a constant). **B.** Shows the derivative of the adaptive fusion penalty, which is used to implement adaptive fusion through local quadratic approximation. The adaptive fusion penalty is modified from SCAD (smoothly clipped absolute deviation) and MCP (minimax concave penalty) functions and like these penalties has a zero derivative far from the origin.

Although we expect that orthology provides some evidence of regulatory similarity, we know that it is not a perfect proxy for functional conservation [22–24]. Some gene interactions may be non-conserved, and in order to minimize bias it would be desirable to identify these interactions and relax the fusion constraints on them. To this end, we developed an adaptive fusion algorithm that attempts to optimize a nonconvex saturating penalty function on differences between fused interactions (Fig 2). Pairs of interactions that are dissimilar even after fusion, which sit in the flat portion of this penalty function, are effectively “unfused,” and no further penalty is incurred as differences in interaction weights grow. Our network procedure strongly favors similarity of fused interactions, and only “unfuses” interactions when their similarity cannot be reconciled with expression data. As a result, the “unfusing” or relaxation of the fusion penalty on certain constraints serves not only to reduce bias in the inferred network weights, but can be interpreted directly as evidence against conservation.

**Fig 3:**
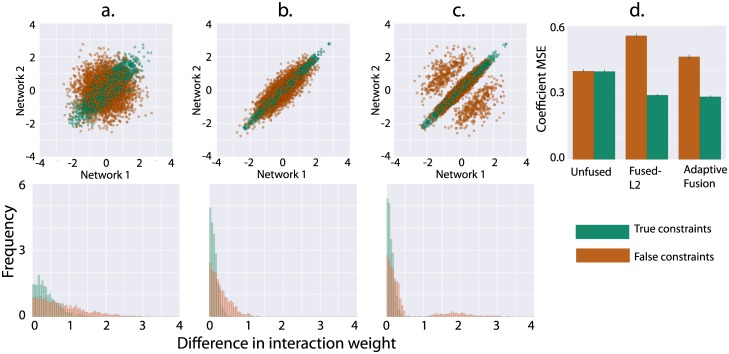
In order to evaluate the performance of adaptive fusion in the presence of non-conserved interactions, we performed a series of simulations inferring networks given a partially corrupted list of orthology mappings (or, orthology mappings that do not represent functional similarity). Networks were generated with 35 TFs by 200 genes, 60% orthology coverage, 40% false orthology coverage, and 30 samples per network. **A.** Top: we plot the interaction weights between pairs of fused interactions in network 1 (x-axis) and network 2 (y-axis) following network inference without fusion (λ_*S*_ = 0). True fusion constraints are generated from pairs of true orthologs, while false fusion constraints derive from a pair of orthologs at least one of which is false. When λ_*S*_ = 0—equivalent to fitting the networks independently—interactions linked by false constraints are uncorrelated with one another, while interactions linked by true constraints are correlated, reflecting their functional similarity. Below we plot the distribution of differences in weights joined by true and false constraints; true constraints have differences on average closer to zero. **B.** shows the weights of interactions when networks are fit using fused-L2. In this condition, interactions bound by both true and false fusion constraints are forced to be very similar across the two species. **C.** With adaptive fusion, many of the false fusion constraints are relaxed. These constraints are no longer forced to be similar, and some of the original structure is restored. **D.** Shows the effect of fused-L2 and adaptive fusion on the accuracy of network recovery, as measured by MSE between the known simulated network weights and the inferred network weights. When fusion is introduced, recovery of the correctly fused part of the network improves, but the bias induced by fused-L2 degrades performance on the incorrectly fused part of the network. With adaptive fusion, the gains on the conserved part of the network are preserved with significantly less loss of accuracy on the non-conserved part of the network. Error bars are 95% confidence intervals on the squared error of coefficients.

We performed a simulation to assess the performance of adaptive fusion in the context of partially conserved networks. We generated synthetic fused networks based on a partial orthology mapping, then randomly assigned the remaining genes unrelated orthologs. These false orthologs gave rise to false fusion constraints, which if enforced would degrade network inference performance. Slightly more than half of the fusion constraints in the generated network were false. We were interested in the ability of adaptive fusion to identify these false fusion constraints, as well as improve the accuracy of the inferred networks by reducing the bias associated with fusion. We verified that solving these partially conserved networks with fused-L2 forced interactions bound by both true and false fusion constraints to be similar (Fig 3b). Fusion led to improved performance on the conserved part of the network and degraded performance on the non-conserved part of the network (Fig 3d, Fused-L2). Applying adaptive fusion with the “a” parameter set to a percentile that roughly matched the fraction of false fusion constraints preserved the performance gains obtained through fusion on the conserved part of the network, while substantially reducing the loss on the non-conserved part of the network. Many of the incorrectly fused interactions were relaxed, allowing these interactions to freely fit the data without bias from a faulty prior. These non-conserved interactions—which have diverged from one another and left the diagonal in Fig 3c—can be identified through inspection of the λ_*S*_ parameter following adaptive fusion, which falls to zero for unfused interactions. This application suggests that adaptive fusion has use as both a tool to reduce bias while retaining many of the benefits of fused regression, and as a method for determining conservation across networks.

### Fused network inference with bacterial data

We used gene-expression data from *Bacillus subtilis* and *B. anthracis* in order to assess performance gains of fused regression on real data. Our *B subtilis* data set consists of 360 time-series and steady-state observations of 4891 genes, 4100 of which are protein coding [25], during the life cycle. Our *B. anthracis* dataset consists of 72 time-series and steady-state observations of 5536 genes comprising data from distinct points in the life cycle and iron-starvation conditions. There were 247 known transcription factors (TFs) in the *B. subtilis* dataset, and 248 TFs in the *B. anthracis* dataset. We obtained 1,870 one-to-one orthologs from Inparanoid [26], 95 of which are transcription factors, which produced 177,650 fusion-constraints between gene interactions within the two species. This number represents only 14.7% of the regulatory interaction matrix in *B. subtilis* and 12.9% in *B. anthracis*.

**Fig 4:**
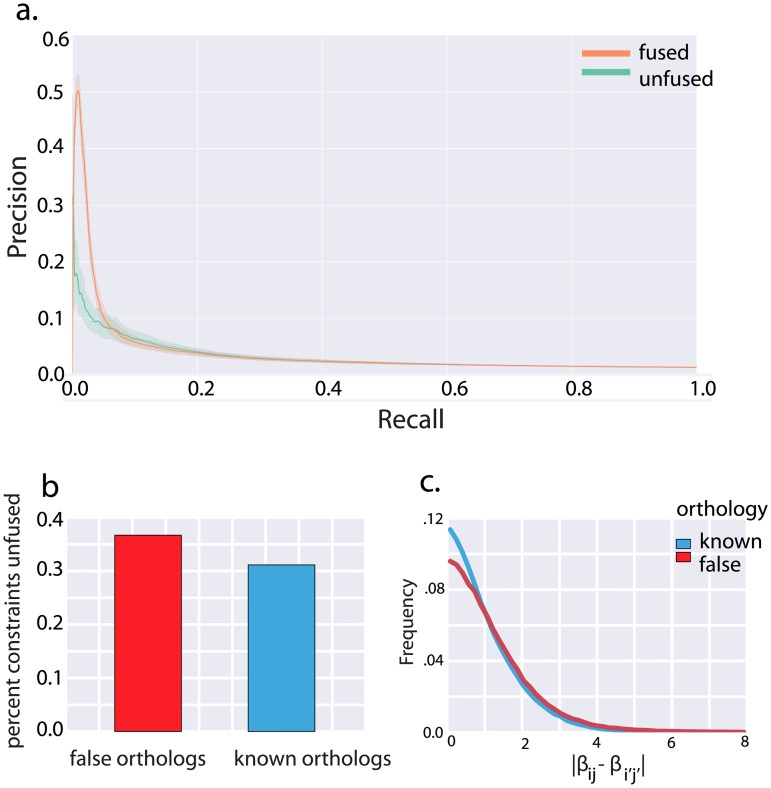
**A** We first optimized λ_*R*_, then performed a 20-fold cross-validation procedure in which subsets of the *B. subtilis* network were fit either independently or fused to the *B. anthracis network*. Each *B. subtilis* network was then evaluated on a gold standard of known interactions, to simulate results of network inference with a small amount of available data in the species of interest (*B. subtilis*) but more data available in a related species (*B. anthracis*). Fusion with λ_*S*_ = 1.0 improved performance, yielding a mean AUPR across the learned networks of 0.0388 vs. 0.0298 fit independently of *B. anthracis*. Plotted is the resulting precision-recall curve, with significant performance gains at low values of recall. We show 95% bootstrap confidence interval. In **B.,C.** we tested adaptive fusion using the same procedure, adding an additional 561 randomly selected additional orthologs. We set the *a* parameter of adaptive fusion equal to 60*t* percentile of the differences in fused interaction weights when networks were fit with λ_*S*_ = 0, so that approximately 40% of constraints were relaxed. This reflected our belief that many of the fusion constraints arising from known orthologies did not necessarily reflect functional similarity, and could be unfused. **B.** Shows the fraction of fusion constraints arising from known orthologies and false random orthologies which were relaxed by adaptive fusion; a larger fraction of constraints known to not reflect functional conservation were relaxed. **C.** After fitting the networks independently, we plot the distribution of the differences in weight of interactions fused by known false fusion constraints and fusion constraints arising from known orthology.

To assess network inference performance, and for use as priors, we used a gold standard of 3,040 known *B. subtilis* interactions with corresponding activation and repression sign. Of these 3,040 priors, 968 had corresponding interactions in *B. anthracis*. Based on our simulation results, we can expect the greatest gains in network-inference performance from fusion when the species of interest has a small number of available conditions, but data is abundant in a related species. However, in order to evaluate performance objectively a gold-standard of known interactions is necessary. As a result, we can only evaluate network recovery for *B. subtilis*, and *B. subtilis* also has the majority of our conditions. In order to simulate the data-poor regime, we subsampled our *B. subtilis* data. We divided our *B. subtilis* data into *k* folds, and then for each fold fit a network to the *B. subtilis* data from that fold alone fused to the entire 72 *B. anthracis* conditions (Fig 4a). Though overall performance is hindered by our subsampling of *B. subtilis* data (a necessary procedure to allow evaluation of networks) we demonstrate marked improvement in learning the *B. subtilis* network when using fused regression (Fig 4a). Notably, these performance gains occur mostly at low values of recall. That is: the highest confidence part of the network is inferred more accurately, with minimal gains for interactions which are more uncertain. Because these interactions are likely to be the focus of followup experiments and validation, gains here are more valuable for prioritizing the order in which interactions are investigated.

#### Adaptive fusion using bacterial data

When we applied adaptive fusion to our bacterial datasets we did not see a significant improvement in network recovery relative to fused-L2. Based on simulation results, we expected that non-conserved interactions weights would be fit more accurately with adaptive-fusion than with fused-L2, but that both algorithms would perform worse than independently fitting the networks on this subset of interactions. It may be that the quality of the independently fit network was too poor in this case for fusion constraints on non-conserved interactions to meaningfully degrade network inference performance. Nevertheless, we were interested in the application of adaptive fusion to identifying neo-functionalized or non-conserved interactions. Essentially, we wanted to identify constraints arising from orthology that does not correspond to functional conservation. However, because we lacked a comprehensive gold standard of known non-conserved interactions between *B. subtilis* and *B. anthracis*, we were forced to indirectly evaluate the performance of adaptive fusion in identifying these interactions.

We obtained a known set of non-conserved interactions by randomly generating orthology mappings between genes not known to be orthologous. These orthology mappings generated false constraints, as in our simulation studies, which are unlikely to reflect any conserved network structure. We used these constraints served as a proxy for the unknown fraction of non-conserved interactions between orthologous genes. We ran adaptive fusion to learn networks for *B. subtilis* and *B. anthracis*, using these constraints along with those generated by known orthology. We confirmed that our injected spurious fusion constraints were unfused at a higher rate than those generated by known orthologs (see Fig 4b & 4c). Although it may seem odd that a large fraction of fake constraints were left intact, we note that biological networks tend to be sparse, so that many of the random fusion constraints are between coefficients with near zero weight (and therefore near zero difference in weight) (Fig 4c). It is unclear to what extent the relatively large fraction of fusion constraints between true orthologs that were unfused reflects an inadequacy of the method, or a relatively high rate of neofunctionalizaton in these organisms.

#### Single species applications

Although the approach we describe was developed with cross-species network inference in mind, the framework of defining fusion constraints on TF-gene interactions expected to be similar is quite general, and can be applied to data from a single organism. Despite the many large-scale collaborations which attempt to make protocols as uniform as possible for comparability between datasets generated by different labs [27, 28] and several methods for removing batch effects [29, 30], there still exists technical and biological variability between many experiments attempting to capture the same or similar experimental conditions especially when experiments employ different experimental platforms. With the advent of RNAseq, for example, microarray based technologies are no longer the dominant assay for genome-wide expression, but a large body of accumulated legacy data remains useful if it can be integrated with more modern techniques.

**Fig 5:**
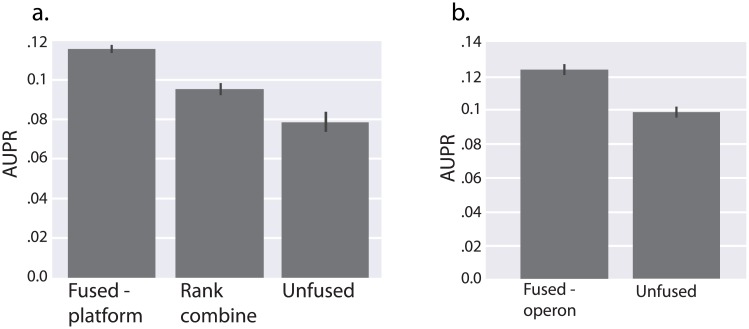
Demonstration of the application of fused-L2 to intra-species network inference *B. subtilis*. In each example, λ_*R*_ is optimized separately without fusion and 10-fold cross validation is used when fitting networks (although, in **A** the gold-standard was not used in fitting the network and did not vary across folds) **A.** We compared the performance of independently fitting our main *B. Subtilis* dataset with two methods for incorporating data from another strain of *B. subtilis*. We evaluated performance on a gold-standard of known interactions. Adaptive fusion outperforms both an independently fitting the first *B. subtilis* dataset and fitting both *B. subtilis* datasets then rank-combining the results, as in Marbach et al. **B** We demonstrate the application of a prior based on operon membership. We generated fusion constraints between pairs of interactions for which both the TF and gene belonged to the same operon respectively. We then held out half of the gold-standard and used it as a prior on individual interactions, as in [33]. We fit the *B. subtilis* network with and without fusion, then evaluated on the remaining gold-standard. In this example, using fusion constraints to enforce a prior based on co-regulation of genes in the same operon improved network inference performance.

Currently, the most widely used approach to combining datasets for network inference is to learn networks from disparate datasets separately, then rank combine the networks as in Marbach et al [20]. We included, along with our main *B. subtilis* dataset, a previously published dataset containing 269 samples covering 104 conditions, obtained using a different tiling microarray (vs custom microarray) and different strain of *B. subtilis* [31]. We compared performance when learning the networks separately and then rank combining (as in [2]) to learning the networks simultaneously using fusion regression, and we show improvement in performance using our fused L2 approach (Fig 5a).

Our approach can also make use of forms of biological data other than RNA expression. Information about the expected similarity of TF-gene interactions can come from knowledge about the promoter region or the structure, for bacteria, of polycistronic transcripts. In bacteria, genes within the same operon are typically under the control of the same promoter [32]. We posited, therefore, that genes within the same operon will be regulated similarly by the same transcription factors. We applied fusion regression by creating fusion constraints between a given transcription factor and genes within the same operon. We also used a subset of the gold-standard as priors by relaxing the λ_*R*_ parameter for these interactions, as in [33], and evaluated on the remaining set of priors. Incorporation of operon-derived fusion constraints in *B. subtilis* improved network recovery (Fig 5b).

### Transcription factor activity estimation integrates into fusion regression approach

**Fig 6:**
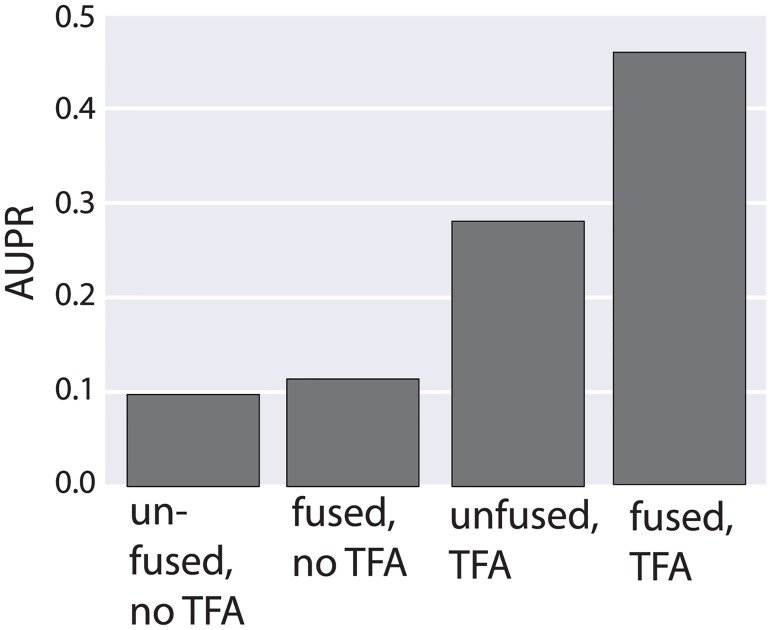
Demonstrates the integration of transcription factor activity (TFA) in fused network inference. The procedure was identical to Fig 5a, except for the additional pre-processing step of transforming transcription factor abundances into an estimate of their activity (see Methods). We then compared performance on the main *B. Subtilis* strain with and without fusion to the second strain, and with and without TFA. TFA outperforms both fused-L2 and unfused inference based on transcription factor abundance, but TFA combined with fusion dramatically outperforms all three methods.

We tested a combination of our fused regression approach with a method for estimating transcription factor activities (TFA). Rather than modeling gene expression using transcription factor mRNA abundance, we fit gene expression as a function of transcription factor activity, as applied to *B. subtilis* by Arrieta-Ortiz et al [4]. TFA activity estimates transcription factor activities that are modulated through mechanisms such as dimerization and interaction with required factors. TFA activity estimates have been shown prior to be better predictors of TF function than expression level alone in several contexts including similar network inference tasks [34] [4]. We estimate TFA based on known regulatory interactions using network component analysis [35]. To test the integration of this approach with our fused regression, we assessed the combination of *B. subtilis* datasets, as in Fig 5a, with the incorporation of TFA estimation. We randomly divided the prior known interactions in half, and used half to learn TFA and to generate priors on network structure. The remaining interactions were reserved as a gold standard for validation. As in previous studies, we observed a marked improvement in network inference when using transcription factor activity (Fig 6). We also obtained AUPR improvement when using fused regression alongside TFA; gains from sharing information across datasets using fused regression were preserved and even enhanced by using TFA.

## Methods

### Statistical approach and background

#### Fused L2

We consider prediction and coefficient estimation problems with *N* observations of *M* dependent variables *y*_1,1_, *y*_2,1_, …*y*_*N*,1_, …, *y*_*N*,2_, …, *y*_*N*,*M*_ and *p* features *x*_*i*,*j*_, *i* = 1, 2, …, *N*, *j* = 1, 2, …, *p*. We begin with a standard linear regression model:

$$y_{i,k}=\sum_{j}x_{ij}\beta_{j,k}+\epsilon_{i}$$ (1)

with errors *ϵ*_*i*_ having mean 0 and constant variance, and predictors *x*_*ij*_ having mean 0 and unit variance. We are interested in the case where *p* > *N*. Many methods have been proposed to deal with the under-constrained case, and have been applied to genomic data [36, 37]. For example, ridge regression penalizes the L2 norm of the coefficients *β*_*i*,*j*_ in order to avoid overfitting [38], and can be thought of as a mean-zero Gaussian prior on the coefficients. More complicated penalties have been developed to represent specific expected or desirable structure in a regression model’s coefficients. For example, Land and Friedman [39] proposed a fusion penalty (or a penalty on the differences between certain interaction weights) which encourages smoothness of the estimated parameter vector. Previous approaches have used fusion penalties to draw statistical strength across multiple regression tasks [40–44]. Price et al. and Bilgrau et al. use a fused ridge estimator for jointly estimating multiple inverse covariance matrices [45, 46]. We take a related approach to these prior works, adding an L2 penalty on the differences between coefficients to the existing ridge penalty in order to incorporate prior knowledge about relationships between input-output pairs:

$$\underset{\beta }{arg min}\sum ||X\beta -{Y||}^{2}+\lambda_{R}{\left||\beta |\right|}^{2}+\lambda_{S}\sum_{\beta_{g,k}\approx \beta_{h,l}}\left|\right|\beta_{g,k}-\beta_{h,l}{\left|\right|}^{2}$$ (2)

where *X*, *Y*, and *β* are matrices, and *β*_*g*,*k*_ ≈ *β*_*h*,*l*_ denotes fusion between entries of *β* (enforcing similarity between model weights across separate datasets). Note that, like ridge regression, this penalty can be thought of as representing a Gaussian prior on the coefficients *β*. In the case where *β* is a column vector, introducing this penalty is equivalent to assuming that *β* is sampled from a multivariate Gaussian with inverse covariance matrix $\sum^{-1}=\lambda_{R}+\sum_{\beta_{g}\approx \beta_{g}}\lambda_{S}\left(1_{g,g}+1_{h,h}-1_{g,h}-1_{h,g}\right)$, where *I* denotes the identity matrix and 1_*i*,*j*_ a matrix of zeros with 1 in its *i*, *jth* entry. In the case of a two-coefficient model with fusion between the coefficients, for example, fused L2 is equivalent to assuming a prior with variance $\left(\lambda_{R}+\lambda_{S}\right)/\left(\lambda_{R}^{2}+2\lambda_{R}\lambda_{S}\right)$ and covariance $\lambda_{S}/\left(\lambda_{R}^{2}+2\lambda_{R}\lambda_{S}\right)$.

#### Solving fused L2 problems using augmented matrices

We begin with the problem of constructing a design matrix to map our problem to that of solving a fused L2 regression problem with a single response variable. We then go on to show that, although the vectorized solution involves solving an impractically large system of equations, under typical biological conditions the structure of constraints allow the problem to be broken up into many smaller subproblems. Key to this approach is the observation that ridge constraints can be incorporated into a least-squares regression problem by appending a scaled identity matrix to the design matrix, and a corresponding number of zeros to the response vector. Similarly, a fusion constraint λ_*S*_(*β*_*i*_ − *βj*)^2^ can be incorporated into a least-squares regression problem by appending a row containing $\sqrt{\lambda_{S}}$ in the *i*th position, $-\sqrt{\lambda_{S}}$ in the *j*th position, and 0s elsewhere to the design matrix, and zero to the response vector. In order to convert an optimization over multiple response variables and multiple sources into an optimization with a single source and response variable, we vectorize as follows: we construct a new design matrix by diagonally concatenating design matrices from relevant regression problems, and create a new response vector by concatenation of corresponding response vectors.

**Fig 7:**
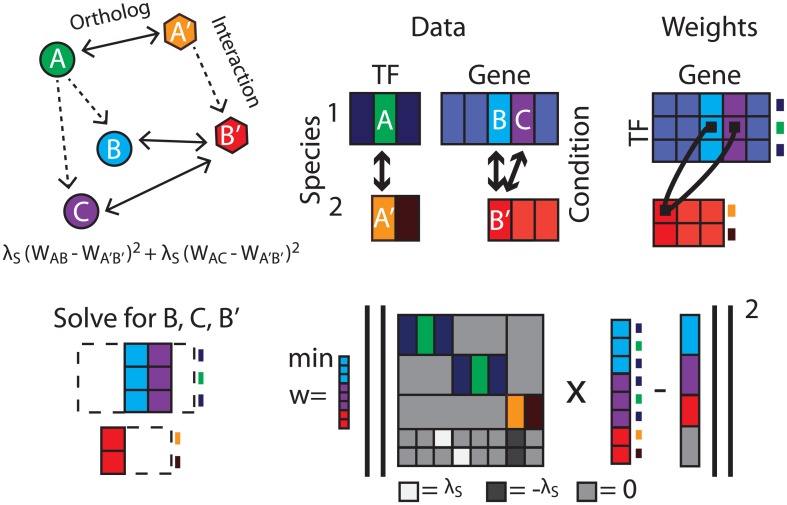
Schematic representation of design matrix construction. Here, the circles and hexagons correspond to different species. Bidirectional arrows represent orthology information and dotted arrows represent putative interactions between TFs and genes. Rectangles under “Data” represent TF × condition matrices of gene expression values in species 1 (top row) and species 2 (bottom row), colored in correspondence with the gene orthology diagram, with bidirectional arrows representing orthology between the two species. Rectangles under “Weights” represent gene regulatory interactions in each species, with lines linking coefficients that are fused due to the orthology information shown to the left. Networks associated with genes in each species can be solved independently unless there exist a fusion constraint constraining coefficients of each gene, or a path of such constraints. When a path does exist, these genes must be solved simultaneously. In this example, genes *B*, *C*, and *B*′ must be solved simultaneously; the lower right corner shows a representation of the design matrix necessary to solve the this fused regression problem.

This is equivalent to the original problem due to the block structure of matrix multiplication. In an ordinary regression problem each response variable can be solved independently, and vectorization is unnecessary. However, in fused regression, we append additional rows to the design matrix that link entries of the interaction weight matrix associated with different response variables (Fig 7). As a result, these linked response variables must be solved simultaneously through vectorization. Two response variables are linked if any of the regulatory weights affecting those genes are linked by a fusion constraint. Two response variables must be solved simultaneously if there is any chain of linked response variables connecting them. However, every other response variable can be solved separately. In biological terms, the regulators of two genes (whether in the same species, or different species) must be solved together if there is a fusion constraint linking those genes’ regulators, or if there is a chain of such constraints. If the networks for a large number of genes are solved simultaneously, the system of equations can quickly become intractable.

In order to avoid this difficulty, we use depth-first search to identify linked columns of each TF expression matrix, then form design and response matrices through vectorization. We can then incorporate fusion constraints as in the case of single-source single response-variable fused regression. In most cases, we have found the direct solution using augmented matrices to be adequate (possible due to the sparse structure of orthology links; only a small number of genes must be solved at once). In the general case, the size of the design matrix is proportional to the number of response variables that must be solved simultaneously. Because the scaling of this algorithm has a complicated dependence on the constraint structure used, a general description of its runtime is difficult. However, in the case of multi-species network inference with one-to-one orthology, the network associated with each pair of orthologous genes requires solving a linear system with approximately twice as many observations and unknowns as the single species case. Linear systems of this size can be solved quickly using standard techniques, and runtime using our bacterial datasets clocks in around thirty minutes. When the size of the groups of genes linked by fusion constraints becomes large (when organisms have a number of many-to-many orthologous blocks), however, the augmented design matrix approach becomes slower and we discuss further optimizations to this scheme below to enable scaling to these regimes.

#### Solving fused L2 problems using iterative solver

To address scaling limitations when many-to-many fusion constraint blocks occur, we developed an iterative solver that uses coordinate-wise descent to solve for solutions corresponding to a sequence of values of fusion penalty weights. As our fused L2 method uses a convex and differentiable penalty function, this approach converges to a global minimizer. Although less efficient than the augmented design matrix approach we developed for cases where fusion constraints are primarily one-to-one or few-to-few, the iterative solver has the advantage of computing a solution path for λ_*S*_ and scaling well across a wider range of biological applications.

On each iteration *t* the iterative solver computes

$$\underset{\beta^{S}}{arg min}\sum_{S\in \{1,2\}}\left|\right|X^{S}\beta^{S}\left(t\right)-Y^{S}{\left|\right|}^{2}+\lambda_{R}\left|\right|\beta^{S}{\left(t\right)||}^{2}+\lambda_{S}\sum_{\begin{array}{l} (g,k)\in orth,\\ (h,l)\in orth \end{array}}\left|\right|\beta_{g,k}^{S}\left(t\right)-\beta_{h,l}^{S}\left(t-1\right){\left|\right|}^{2}$$ (3)

Note that this is almost identical to eq 5, but now the network *β* is a function of the iteration number *t*. On each step, we compute *β*s that minimize a penalized cost function where the fusion penalties encourage similarity between a parameter and its fused-to parameter from the previous iteration’s solution. This process is iterated until the estimated *β*s converge. Because each iteration reduces the error between *β*(*t*) and *β*(*t* − 1), and because *β*(*t*) = *β*(*t* − 1) is the globally optimal solution, this process must eventually converge to the same network as eq 2. Although we have not produced bounds on the convergence rate, which also depends on the structure of constraints, in practice a small number of iterations (∼10) are necessary.

#### Fusion and regularization path

Optimizing over both parameters, λ_*R*_ and λ_*S*_, is computationally prohibitive and we opted to test a heuristic where we optimize the two parameters separately. Our procedure first optimized λ_*R*_ with λ_*S*_ = 0, then optimized λ_*S*_ using this value of λ_*R*_. This procedure is guaranteed to achieve the best unfused solution in the case when λ_*S*_ is constrained at 0. To optimize λ_*R*_ we use cyclical coordinate descent algorithms from the ‘glmnet’ package [47] to compute a ridge regularization path. We use cross validation to select the optimal λ_*R*_ parameter from this path, selecting the λ_*R*_ which minimizes the average error of prediction on a leave out set across cross validation folds. Following selection of λ_*R*_, we search for optimal λ_*S*_ by computing the solution path from the iterative solver (using the sequence of successive model weights) again using cross validation to select the optimal parameter. Note that both parameters are chosen without reference to the gold standard, which is used in a separate evaluation of network quality.

#### Adaptive fusion

Fusion constraints penalize dissimilarity between interactions thought to be analogous based on *a priori* knowledge. For example, orthology can be used to predict which interactions will be similar across species. With an L2 fusion penalty, interaction weights which differ from each other by a large amount are excessively penalized, which effectively ensures that fused interactions are assigned similar weights. This will be inappropriate for interactions which are identified based on orthology as being analogous, but which are no longer similar due to evolutionary changes. We propose a saturating penalty that is relaxed once differences in weights grow beyond a certain point (interactions which appear to be very different based on the data are effectively unfused). A related problem has been studied in the context of LASSO regularization, where it was shown by Fan and Li that using a saturating penalty retains many of LASSO’s desirable properties while removing its bias towards model weights of 0 [21]. They further showed that, although the resulting loss-function is non-convex, good results can be obtained with a local quadratic approximation of gradient descent. Several saturating penalties, such as SCAD [21] and MCP [14], have been discussed in the context of sparse regression. We introduce a modified form of MCP to the problem of penalizing differences between fused coefficients. The principal difference between the penalty we adopt and SCAD/MCP is that both of these penalties are L1 like at the origin, producing sparse solutions. Some network inference approaches use L1 penalties to produce sparse networks, on the basis that biological networks are thought to be sparse. However, as we are penalizing differences in interaction weights, rather than the weights themselves, there’s no reason to assume that most differences will be exactly zero, and an L2 penalty—equivalent to an assumption that the differences between fused coefficients are Gaussian distributed—may be more appropriate.

We use a penalty on the difference between fused coefficients *θ* which is L2 like at the origin and saturates at *θ* = *a*. Written in terms of its derivative, the penalty $p_{\lambda ,a}^{\prime }$

$$p_{\lambda ,a}^{\prime }\left(\theta \right)=\left\{\begin{array}{cc} \lambda \theta & \text{if}\;\theta \leq \lambda \\ \text{max}(\lambda (2a-\theta ),0) & \text{if}\;\theta >a \end{array}\right.$$ (4)

As in [21], we solve using iterative local quadratic approximation. Specifically, *β*^*S*^(*t*) is the network on iteration *t*. For each fused $B_{g,k}^{S_{1}}\approx B_{h,l}^{S_{2}}$ we define:

θ(0)=0 (5)

$$\theta \left(t\right)=|B_{g,k}^{S_{1}}-B_{h,l}^{S_{2}}|$$ (6)

and introduce a fusion constraint $\lambda =\frac{p^{\prime }\left(\theta \left(t\right)\right)}{2\theta (t)}$

*β*^*S*^(*t* + 1) is obtained by fitting the ridge-fused model with fusion constraints given by the above λ_*S*_. This is useful because all our penalties can be treated as L2 and therefore retain the properties of ridge regression, and can be solved using the fused L2 algorithm we develop.

Our adaptive penalty function introduces, in addition to regularization and fusion penalty weights λ_*R*_ and λ_*S*_, an unknown parameter *a*. We could employ grid search using cross-validation to search for the best parameters, but for many data sets, this can be computationally expensive. Moreover, we are primarily interested in using this saturating penalty as a way of testing the hypothesis that conservation in GRNs can be predicted based off of known similarities between genes. Therefore, we propose setting *a* based on the distribution of differences between fused weights from independently fit networks. These networks can be fit without fusion, prior to the adaptive fusion procedure. The choice of which percentile of this distribution to use for *a* represents the working hypothesis for the fraction of fused interactions which should be unfused.

### Biological application

Although our approach is generalizable to a wide variety of multi-source network inference problems, we begin with the concrete example of network inference in two related species. Our approach to multi-species network inference is based on the hypothesis that gene regulation in related species is governed by similar but not necessarily identical gene regulatory networks, due to conservation of function through evolution. We represent conservation of network function by introducing constraints into the objective function for network inference that penalize differences between the weights of regulatory interactions believed to be conserved. These constraints favor the generation of similar networks for related species, and in the generally under-constrained regime of network inference can improve the accuracy of network recovery. We then go on to introduce a method to test the assumption of conserved network structure, and to relax the associated constraints on pairs of interactions for which the data does not support conservation. Finally, we demonstrate the flexibility of the method by using fusion constraints based on operon membership to improve network inference performance.

#### Approach overview

For a high-level view, we summarize our approach:

**Algorithm 1** Network inference using fused regression

 load expression data

 load orthology

 create priors and fusion constraints

 partition gold standard into training and leave-out

 generate TFA matrices using gold standard training set

 set *a* if using adaptive fusion

 **for** *k* in folds **do**

  partition expression data into training and leave-out set

  λ_*R*_ parameter selection using training set

  λ_*S*_ parameter selection using training set

  run fused regression

  return PRC and ROC curves using leave-out gold standard

 **end for**

 average PRC / ROC curves over folds

#### Gene regulatory network

We model the transcription rate of each gene as a weighted sum of transcription factor expression, and seek to identify the identities and regulatory weights of these TFs. This formulation matches that of the existing *Inferelator* algorithm, which models the linear dynamics of gene expression [48]. Our primary data for learning gene regulatory networks is expression data, consisting of both time-series and steady state experiments. The rate at which *x*_*i*_, the observed mRNA expression of gene *i*, changes, is governed by degradation of existing transcripts with rate *α* plus a linear combination of transcription factor (TF) expressions.

$$\frac{d}{dt}x_{i}=-\alpha_{i}x_{i}+\sum \beta_{i,j}x_{j}$$ (7)

where *β*_*i*,*j*_ represents the weight of TF *j* on gene *i*, and *α* is the decay rate of gene *i*. We fix the decay rate *α* for all genes, and set it assuming a time-constant of 10 minutes [49, 50], as in [33]. Let *x*_*i*_(*t*) be the expression of gene *i* at time *t*. Given time-series data on the expression of gene *i* at timepoints *t*_*k*_ and *t*_*k*+1_, we can approximate the rate of change of *x*_*i*_ as $x_{i}^{\prime }\left(t_{k}\right)=\frac{x_{i}\left(t_{k+1}\right)-x_{i}\left(t_{k}\right)}{t_{k+1}-t_{k}}$. We treat steady-state data as having a derivative of zero.

For timeseries conditions this gives us, for each gene *i* and time *t*_*k*_ an equation

$$\frac{x_{i}\left(t_{k+1}\right)-x_{i}\left(t_{k}\right)}{t_{k+1}-t_{k}}+\alpha_{i}x_{i}\left(t_{k}\right)=\sum \beta_{i,j}x_{j}\left(t_{k}\right)$$ (8)

where *j* ≠ *i*

And for steady state conditions

$$\alpha_{i}x_{i}\left(t_{k}\right)=\sum \beta_{i,j}x_{j}\left(t_{k}\right)$$ (9)

Both kinds of conditions can be included in the set of equations to be solved. We can summarize these equations in matrix form as

Y=Xβ (10)

where *Y* is the gene expression matrix, *X* is the TF expression matrix, and *β* is the regulatory weights we are interested in learning. We are interested in learning *β*, the matrix representation of the gene regulatory network, where the weight in a given position represents the regulatory weight of a TF on a gene. Positive weights represent activation, negative weights represent repression, and 0 weights represent the absence of an interaction. The matrix *β* can be solved using linear regression. Because there are typically far fewer conditions than possible regressors (TFs), we introduce a ridge regularization constraint with weight λ_*R*_ and solve

$$\underset{\beta }{arg min}||X\beta -Y_{2}{\left|\right|}^{2}+\lambda_{R}{\left||\beta |\right|}^{2}$$ (11)

This is similar to the formulation used in the *Inferelator* algorithm, except that we use an *L*2 penalty in place of the more standard *L*1 penalty. Although *L*1 penalties have the theoretical appeal of sparsity—which matches our assumption of sparsity in biological networks—we are primarily interested here in ranking interactions, rather than recovering a specific network. Moreover, *L*2 penalized regression followed by thresholding can be competitive with *L*1 penalized regression for support recovery [51]. Although our approach can be easily modified to use an *L*1 penalty, we did not observe performance gains when doing so, and the version presented here is in some ways conceptually simpler (with the combined penalty function being equivalent to a multivariate Gaussian prior).

Because a transcription factor’s raw abundance is not always a good predictor of its influence on its gene targets’ expression, previous network inference methods have attempted to estimate transcription factor activities prior to network inference. When there exists a set of prior known interactions, we are able to estimate transcription factor activity (TFA) using network component analysis [35], as in [4, 34], and use TFA as explanatory variables instead of transcription factor expression.

#### Fused gene regulatory networks

Information about the partially conserved structure of gene regulation is introduced through the incorporation of constraints into the above regression formulation. These constraints penalize differences between interaction weights in the networks of multiple species that are expected to be similar based on prior biological knowledge. We can then solve the penalized regression problems simultaneously, in order to obtain a gene regulatory network (GRN) for each species.

Consider the case of organisms *A* and *B*, governed by GRNs *β*^*A*^ and *β*^*B*^ (the following approach applies equally well to more than two species—where the set of constraints is the enumeration of all constraints between pairs of species). When TF *g*^*A*^ in organism *A* and TF *h*^*B*^ in organism *B* are orthologs, and gene *k*^*A*^ and *l*^*B*^ are orthologs, then we expect that the *g*^*A*^ → *k*^*A*^ interaction weight should be similar to the *h*^*B*^ → *l*^*B*^ interaction weight, and we introduce a fusion constraint between these analogous interactions. This is shown schematically in Fig 1. In terms of the above regression formulation, we expect that $\beta_{g,k}^{A}\approx \beta_{h,l}^{B}$, and include a penalty term $\lambda_{S}p\left(\beta_{g,k}^{A}-\beta_{h,l}^{B}\right)$ in the quantity being minimized in order to encourage similarity.

The function *p*(*x*) controls the shape of the penalty function, while scalar λ_*S*_ controls the overall scaling of the penalization of differences between fused coefficients. λ_*S*_ controls the tradeoff between fitting the expression datasets individually and producing a set of networks that conform to evolutionary prior knowledge. This gives us the final equation to be minimized:

$$\underset{\beta^{S}}{arg min}\sum_{S\in \{1,2\}}\left|\right|X^{S}\beta^{S}-Y^{S}{\left|\right|}^{2}+\lambda_{R}\left|\right|\beta^{S}{\left|\right|}^{2}+\lambda_{S}\sum_{\begin{array}{l} (g,h)\in orth,\\ (k,l)\in orth \end{array}}p\left(\beta_{g,k}^{S_{1}}-\beta_{h,l}^{S_{2}}\right)$$ (12)

where the second sum is over pairs of interactions with fusion constraints.

As an example of how this formulation allows pooling of data across multiple sources, consider the case where there is a one-to-one orthology between the species being considered (ie different cell-lines of the same organism). The choice of λ_*S*_ allows one to interpolate between fitting each network independently (λ_*S*_ = 0) and pooling data together as if it came from one source (λ_*S*_ = inf). In the case of cross-species network inference, there is unlikely to be a complete one-to-one orthology. However, because we constraints the similarity of individual interactions, rather than on the networks as a whole [52, 53], we can pool some information across species even when a small fraction of genes have orthologs.

#### Simulated data

We generate simulated data to evaluate the ability of our fused L2 approach to learn the true network and to show that sharing information between similar but not identical data sources results in more accurate network recovery. Generation of simulated data begins with the production of random orthology mappings. We produce orthology by pairing random genes until a specified fraction have been assigned orthologs. This process is carried out separately for TFs and non-TF genes, so that TFs and non-TF genes are never assigned to be orthologous. We then produce a pair of random networks (*β*^1^ and *β*^2^) as follows: for each unfilled entry in *β*^1^ or *β*^2^, we enumerate the set *C* consisting of the entry along with every entry in either matrix to which it is fused. We set a sparsity rate and with probability equal to this sparsity rate, we assign every entry in *C* to be 0, otherwise we sample a value v∼N(0,1) and independently assign each entry in *C* to $v+N(0,\sigma_{f}^{2})$. *σ*_*f*_ is a parameter that controls the distribution of differences in the values of fused coefficients, so that the nonzero coefficients of *β*^1^, *β*^2^ are distributed as $N(0,1+\sigma_{f}^{2})$.

Given a network *β*, we generate *N* samples of gene expressions at two timepoints. The condition by gene expression matrix for timepoint one, *Y*_*T*1_, is sampled randomly from a multivariate Gaussian distribution with identity covariance matrix. *X*_*T*1_ is the TF expression sub-matrix of *Y*_*T*1_, and consists of columns of *Y*_*T*1_ that correspond to TFs. Treating the decay rate as 0, the gene expression matrix at timepoint two, *Y*_*T*2_ is sampled as *Y*_*T*2_ = *Y*_*T*1_ + *βX*_*T*1_ + *ϵ*, where *ϵ* is a Gaussian noise term. This process is carried out separately for each network. Following generation of simulated data, we may introduce error into the orthology mapping. This can take the form of discarding a specified fraction of true orthologies (governed by a false-negative rate), by introducing random false orthologies (governed by a false-positive rate), or by adding Gaussian noise so that fused interactions are not identical (described above). For convenience, the false-positive rate is specified in units of the number of true orthologs, and not the number of possible orthologs. The list of priors can in a similar fashion be manipulated to include false positives and false negatives.

#### Ranking regulatory hypotheses

In previous work, betas were rescaled as to form a matrix of confidence scores *S* as follows

$$S_{i,j}=\frac{\sigma_{\text{full}\;\text{model}\;\text{for}\;y_{j}}^{2}}{\sigma_{\text{full}\;\text{model}\;\text{for}\;y_{j}\;\text{without}\;\text{predictor}\;i}^{2}}$$ (13)

Computing residuals with respect to the data alone would disregard information gained through fusion, because certain interactions may be large due to fusion, rather than their individual explanatory power. Instead, we used an approximation

$$S_{i,j}=\frac{\sigma_{\text{full}\;\text{model}\;\text{for}\;y_{j}}^{2}}{\sigma_{\text{full}\;\text{model}\;\text{for}\;y_{j}}^{2}+\beta_{i,j}^{2}\times var\left(TF_{j}\right)}$$ (14)

#### *B. subtilis* and *B. anthracis* data and orthology

We used a dataset collected for PY79, a derivative of strain 168, available on GEO with accession number GSE67023, and a dataset using BSB1, another derivative of strain 168, available at GEO with accession number GSE27219. We used two datasets for *B. anthracis*, transcription profiling during iron starvation (E-MEXP-2272 on ArrayExpress), and time series over the life cycle (E-MEXP-788 on ArayExpress). We ran Inparanoid to obtain orthology mapping for *B. subtilis* and *B. anthracis* [26] To evaluate our approach, we compare with a set of experimentally validated regulatory interactions from SubtiWiki [54].

## Discussion

Gene expression data, such as microarray or RNA seq, provide information about the relationship between genes by allowing an experimenter to measure correlations in expression value over time or across conditions. Many sources of information—such as the knowledge that two genes are related through orthology or belong to the same operon—provide additional information about the relationships between these gene-gene relationships. For example, two genes that belong to the same operon are likely to have a similar set of regulators [32], but knowing that two genes are members of a polycistronic transcript does little to inform the identity (strength, sign) of those regulators. Meta-information about the structure of gene regulatory networks, specifically which pairs of interactions are *a priori* likely to be similar to one another, can provide a powerful set of constraints to improve network inference performance [10, 55]. We present a general framework for gene regulatory network inference that incorporates this meta-information—termed fusion constraints—and apply the technique to the problem of simultaneous inference of regulatory networks in multiple species (*B. subtilis* and *B. anthracis*), as well as to the problems of combining data from multiple experimental platforms and information about operon structure.

A number of existing approaches have applied fused regression to related problems in network inference. TreeGL applied fused LASSO to the problem of estimating partial correlations of gene expression in a breast cancer dataset consisting of multiple cell types [52]. They imposed fusion between the coefficients of models learned for different cell types whenever there was an edge between those cell types in a genealogy graph describing the cancer cells’ development. A similar approach was recently applied to the problem of inferring TF to gene regulatory weights from data sources describing multiple environmental conditions in *E. Coli*, *Mycobacterium tuberculosis*, and *Mus musculus* [53]. In this formulation, networks associated with each data source were fit simultaneously, with an *L*1 constraint on the similarity of (arbitrarily ordered) adjacent networks. In both of these approaches, fusion was restricted to be between corresponding entries of the coefficient matrices. In terms of our formulation in the cross-species case, this is equivalent to the constraints that would be generated by a one-to-one orthology mapping between species, limiting to the (typical) case where orthology is partial or not one-to-one. By optimizing a more general objective function—in which fusion constraints can be placed on arbitrary pairs of coefficients associated with otherwise unrelated regression tasks—we can extend this work to the cross-species case.

*B. subtilis* and *B. anthracis* are distantly related bacterial species with limited gene orthology. Nevertheless we show that network recovery in *B. subtilis* can be improved through the inclusion of expression data from *B. anthracis*. Many previous methods for cross-species network inference operate on the conserved subset of orthologous genes [56]. This assumption may be appropriate with very closely related species, but could not be applied in this domain, where a large fraction (62% and 67%) of the *B. subtilis* and *B. anthracis* genomes do not have clear orthologs. Our method, in contrast, can obtain improvements in network inference performance even when the conserved subset of genes is small.

This approach is particularly interesting in light of the diversity of important model organisms used in modern biology. Different model systems provide different advantages and disadvantages for experimental design [57], but in many cases work in less used systems is hampered by lack of available data. Our intent is to provide a principled method for combining data from diverse sources, so that results in specialized systems can be integrated with data from well studied organism.

Although it is important to take advantage of the similarities of related organisms for generating improved models of gene regulation, it is also critically important to understand how systems differ from one another. Existing approaches to the genome-wide testing of the assumption that orthologous genes have similar regulators learn regulatory networks separately, then compare to identify conservation [58]. Because network inference is typically under-constrained, fitting a network that describes a particular set of experimental observations amounts to sampling a single network from a large set of networks that fit the data equally (or almost equally) as well. As a result, the existence of a difference between corresponding regulatory interactions in a pair of experimentally derived networks is weak evidence that a difference truly does exist. Uncoupled global network inference algorithms are a very weak tool for uncovering evolutionary divergence. Our method explicitly favors recovering networks for which evolutionarily corresponding interactions are similar. As a result, the failure to obtain networks that confirm evolutionary conservation is much more direct evidence that the networks have truly diverged.

Although fused regression allows more accurate identification of pairs of non-conserved interactions, the weights obtained for these interactions will be biased towards one another. We have described a method—adaptive fusion—that attempts to address this bias by simultaneously learning the networks and constraint weights. This method is based on minimizing a saturating penalty function on fusion constraints, similar to a class of penalties that have been developed to minimize bias in regularized regression [14, 21]. The result of adaptive fusion is both a network and a new set of fusion constraints, the weights of which can be interpreted as describing conservation structure across the networks. For the multiple species case, relaxation of fusion constraints represents orthologs which do not share similar interactions presumably due to evolution of regulatory circuitry [59]. When jointly learning networks describing processes in different cell lines, this may identify interesting context-specific behavior. Genes may be fused together on the basis of similar binding sites or chromatin features, and the relaxing of the fusion penalty indicates divergence of gene function.

Because our model shares its basic assumptions about the role of transcription factors in gene expression dynamics with models developed for single-species network inference, we are able to leverage techniques developed for the single-species estimation of transcription factor activity [34]. The performance gains of this additional step in the cross-species case are significant. Our approaches—fused L2 and adaptive fusion—represent a very general framework for simultaneous network inference and the incorporation of structured biological priors. These priors—incorporated into our method as fusion constraints—allow the use of rich sources of biological knowledge, such as orthology and operon structure, which have informed experimental design, but are typically not incorporated into genome wide network inference algorithms. By accommodating the simultaneous inference of multiple related networks, we can improve network inference performance by allowing the efficient reuse of data from similar, but not necessarily identical, sources. A method for pooling data from multiple sources holds the promise of vastly expanding the quantity of data available for analysis, particularly in less commonly used model systems. At the same time these methods allow us to test our assumptions on how similar biological systems relate to one another, by allowing us to rule out conservation in a principled way, and at the genome-wide scale.
